# A current view on long noncoding RNAs in yeast and filamentous fungi

**DOI:** 10.1007/s00253-018-9187-y

**Published:** 2018-07-04

**Authors:** Petra Till, Robert L. Mach, Astrid R. Mach-Aigner

**Affiliations:** 1 Christian Doppler Laboratory for Optimized Expression of Carbohydrate-Active Enzymes, Institute of Chemical, Environmental and Bioscience Engineering, TU Wien, Gumpendorfer Str. 1a, 1060 Vienna, Austria; 2 Institute of Chemical, Environmental and Bioscience Engineering, TU Wien, Gumpendorfer Str. 1a, 1060 Vienna, Austria

**Keywords:** Long noncoding RNA, lncRNA, Yeast, *Saccharomyces cerevisiae*, *Schizosaccharomyces pombe*, *Trichoderma reesei*

## Abstract

Long noncoding RNAs (lncRNAs) are crucial players in epigenetic regulation. They were initially discovered in human, yet they emerged as common factors involved in a number of central cellular processes in several eukaryotes. For example, in the past decade, research on lncRNAs in yeast has steadily increased. Several examples of lncRNAs were described in *Saccharomyces cerevisiae* and *Schizosaccharomyces pombe*. Also, screenings for lncRNAs in ascomycetes were performed and, just recently, the first full characterization of a lncRNA was performed in the filamentous fungus *Trichoderma reesei*. In this review, we provide a broad overview about currently known fugal lncRNAs. We make an attempt to categorize them according to their functional context, regulatory strategies or special properties. Moreover, the potential of lncRNAs as a biotechnological tool is discussed.

## Introduction

Pervasive transcription including intergenic and antisense regions evolved as a common feature in higher and lower eukaryotes (David et al. 2006; Dutrow et al. 2008; Nagalakshmi et al. 2008; Wilhelm et al. 2008). A very heterogeneous group of noncoding transcripts are the long noncoding RNAs (lncRNAs). They are distinguished from small RNA species upon their size of > 200 nt (Djebali et al. 2012; Kapranov et al. 2007), rather than upon any other property. The presence of a poly(A)-tail as well as a 5′-methylguanosine cap or post-transcription splicing events are optional features (Djebali et al. 2012; Kapranov et al. 2010; Yin et al. 2012). More common characteristics of lncRNAs are poor conservation and expression at low levels compared to protein encoding genes (Derrien et al. 2012; Djebali et al. 2012). Furthermore, lncRNAs often fold into complex, high ordered structures (Mercer and Mattick 2013). Transcripts targeted by degradation are also assigned to the lncRNAs (Schulz et al. 2013; van Dijk et al. 2011; Wyers et al. 2005; Yin et al. 2012). Anyhow, it should be considered that the discrimination between lncRNAs and coding transcripts is vague. Several not annotated transcripts, which were initially predicted to lack a protein encoding capacity, were found to be associated with the polyribosomes as they contain small open reading frames (Smith et al. 2014). The modes of action and also the processes, in which lncRNAs are involved in, are manifold. Their strategies range from physical interference with the transcription of adjacent or overlapping sense or antisense-oriented target genes *in cis*, over nucleosome repositioning, histone modifications or the recruitment of chromatin remodelling factors, to *trans* interactions with proteins, DNA or other RNAs, in some cases providing a scaffold for the attachment of multiple factors (reviewed in Ponting et al. 2009). Predominantly, lncRNAs act as repressors, yet also examples for positive regulation of the target genes have been reported (Krishnan and Mishra 2014).

Most lncRNAs were described in human and other mammals (Derrien et al. 2012; Fantom Consortium 2005; Hon et al. 2017; Iyer et al. 2015). In the last years, also the number of lncRNAs identified in the well-studied budding yeast *Saccharomyces cerevisiae* and model fission yeast *Schizosaccharomyces pombe* has rapidly increased (reviewed in (Niederer et al. 2017; Yamashita et al. 2016). In ascomycetes, such as *Neurospora crassa* and *Magnaporthe oryzae,* the physical presence of lncRNAs was reported (Cemel et al. 2017; Jain et al. 2017). And just recently, the first functional characterization of a lncRNA in the filamentous fungus *Trichoderma reesei* was achieved (Till et al. 2018). In this review, we give an overview about relevant research on fugal lncRNAs. Attempts for categorizing them are discussed, and outstanding examples are presented in more detail.

## The challenge of classifying lncRNAs

In contrary to protein encoding genes, lncRNAs do not form large homologous families (Ponting et al. 2009). Thus, the attempt to classify of lncRNAs is a challenging task. Examples of thoroughly described lncRNAs identified in different fungi are listed in Table 1. The categorization was made on a *cis* or *trans* mode of action, the functional context, regulatory mechanism or special properties. Anyway, there are hardly any overlaps of the groups formed by the different strategies of classification. Different regulatory mechanisms are followed by lncRNAs involved in the same category of cellular processes, and also, special properties are not necessarily assigned to a functional context. Also, a *cis* or *trans* mode of action is not consistent with the function or mechanism. Conclusively, at least at the current state of knowledge, a generally valid categorization of fungal lncRNAs is rather pointless. Instead, we favour grouping by means of different criteria, consequently tolerating multiple assignments of some fungal lncRNAs.

**Table 1:** Described lncRNAs in fungi

Name	Fungus	Regulated gene	*cis/trans*	Cellular process	Regulatory mechanism	Special properties	Reference
*SRG1*	*S. cerevisiae*	*SER3*	*cis* (s)	Serine biosynthesis	Transcription interference		Martens et al. (2004)
*GAL10* lncRNA	*S. cerevisiae*	*GAL10, GAL1*	*cis* (as), *cis* (s)	Galactose utilization	Histone modification, silencing	Unstable (decapping)	Houseley et al. (2008)
*GAL4* lncRNA	*S. cerevisiae*	*GAL4*	*cis* (as)	Galactose utilization	Unknown		Geisler et al. (2012)
*nc-tgp1*	*S. pombe*	*tgp1*	*cis* (s)	Phosphate metabolism	Transcription interference by occlusion of Pho7 (TF)	Unstable (MDED)	Ard et al. (2014)
*prt* / *nc-pho1*	*S. pombe*	*pho1*	*cis* (s)	Phosphate metabolism	Transcription interference by occlusion of Pho7 (TF) (RNAi-mediated silencing)	Unstable (MDED)	Chatterjee et al. (2016), Shah et al. (2014)
*prt2*	*S. pombe*	*pho84,* (*prt/pho1*)	*cis* (s)	Phosphate metabolism	Unknown		Garg et al. (2018)
*HAX1*	*T. reesei*	Cllulase genes	*trans*	Cellulose metabolism	Interplay with Xyr1 (TF), details in preparation	3 isoforms, activation	Till et al. (2018)
*CDC28* asRNA	*S. cerevisiae*	*CDC28*	*cis* (as)	Osmostress	Chromatin remodelling	Activation	Nadal-Ribelles et al. (2014)
*SPNCRNA.1164*	*S. pombe*	*atf1*	*trans*	Oxidative stress	Unknown	Activation	Leong et al. (2014)
mlonRNA	*S. pombe*	*fbp1*	*cis* (s)	Glucose starvation	Chromatin remodelling		Hirota et al. (2008)
*ncASP3*	*S. cerevisiae*	*ASP3*	*cis* (s)	Nitrogen starvation	Histone modification, chromatin remodelling	Maintaining attended status	Huang et al. (2010)
*IRT1*	*S. cerevisiae*	*IME1*	*cis* (s)	Meiosis	Transcription initiation block, histone modifications		van Werven et al. (2012)
*RME2*	*S. cerevisiae*	*IME4*	*cis* (as)	Meiosis	Transcription elongation block		Hongay et al. (2006)
*RME3*	*S. cerevisiae*	*ZIP2*	*cis* (as)	Meiosis	Transcription elongation block		Gelfand et al. (2011)
meiRNA	*S. pombe*	Meiosis-specific genes	*cis* ?	Meiosis	RNA-protein IA, inhibition of Mmi1, RNAi	2 Isoforms, activation	Watanabe and Yamamoto (1994)
*SUT169*	*S. cerevisiae*	*SPS100*	*cis* (as)	Sporulation	Regulation of mRNA length and stability	Activation	Huber et al. (2016)
*ICR1*	*S. cerevisiae*	*FLO11*	*cis* (s)	Cell-cell adhesion (filament formation)	Promoter occlusion, silencing		Bumgarner et al. (2009)
*PWR1*	*S. cerevisiae*	*ICR1* (*FLO11*)	*cis* (as)	Cell-cell adhesion (filament formation)	Transcription interference		Bumgarner et al. (2009)
pHO-lncRNA	*S. cerevisiae*	*HO* genes	*cis* (s)	Mating type interconversion, re-entering cell-cycle	Nucleosome repositioning		Yu et al. (2016)
*TERRA*	*S. cerevisiae*	Telomeric DNA	?	Telomere replication	Regulation of telomerase activity, heterochromatin formation, scaffold		Luke et al. (2008)
Telomerase RNA, *TLC1*	*S. cerevisiae*	Telomeric DNA	?	Telomere replication	Scaffold for telomerase complex, telomere template	Ribonucleo-protein	Zappulla and Cech (2004)
Telomerase RNA *TER1*	*S. pombe*	Telomeric DNA	?	Telomere replication	Scaffold for telomerase complex, telomere template	Ribonucleo-protein	Leonardi et al. (2008)
*SUT457*	*S. cerevisiae*	12 genes	*trans* ?	Telomere control	RNA-DNA IA, details unknown	Activation	Kyriakou et al. (2016)
*PHO84* antisense transcripts	*S. cerevisiae*	*PHO84*	*cis* (as), *trans*	Cell aging, silencing	Histone modification	Unstable (CUTs)	Camblong et al. (2007)
*TY1*	*S. cerevisiae*	Ty1 retro-transposon	*trans*	Transposon silencing	Unknown	Unstable (XUTs)	Berretta et al. (2008)
XUTs, screening	*S. pombe*	Various	*cis* (as), trans	Meiosis, others?	Unknown	Unstable (XUTs)	Wery et al. (2018)
NUTs, screening	*S. cerevisiae*	Various	*cis* (as/s)	Silencing	Transcription interference, antisense repression	Unstable (NUTs)	Schulz et al. (2013)
NUTs, screening	*S. pombe*	Various	?	TGS	Histone modification, nucleosome repositioning, protein recruitment	Unstable (NUTs)	Marina et al. (2013)

*as* antisense, *CRF* chromatin remodelling factors, *IA* interaction, *intra* intragenic, *MDED* Mmi1-directed exosome degradation, *s* sense, *TGS* transcription gene silencing, *TF* transcription factor

### *Cis* and *trans* acting lncRNAs

Most lncRNAs described in yeast act in *cis*. This means that they regulate the expression of genes at proximal locations on the same chromosome, whereas *trans*-acting lncRNA affect either distal loci or the same locus but located on a homologue chromosome. Some *cis*-acting lncRNAs were demonstrated to act strictly in *cis* because *trans*-expression in diploids or ectopic expression in a lncRNA-deleted background results in a loss of function of the lncRNA. Among those are the *CDC28* antisense lncRNA (Nadal-Ribelles et al. 2014), *prt* (Shah et al. 2014), pHO-lncRNA (Yu et al. 2016), *ICR1* and *PWR1* (Bumgarner et al. 2009) and *RME2* and *RME3* (Hongay et al. 2006). In contrast to this, for some lncRNAs initially described as *cis*-acting factors, the function was shown to be position independent. One example for this is the *PHO84* antisense transcript, which belongs to the group of cryptic unstable transcripts (CUTs) and triggers silencing of its sense gene *PHO84* in *S. cerevisiae* in aged cells (Camblong et al. 2009; Camblong et al. 2007).

Examples for per se *trans*-acting lncRNAs in fungi are rare. In the budding yeast *S. cerevisiae*, a *trans*-mechanism was stated for the stable unannotated transcript (SUT) *SUT457,* which physically interacts with 12 genes essential for telomere organization and homeostasis (Kyriakou et al. 2016)*.* Another example is *SPNCRNA.1164*, a regulator of *atf1* expression in response to oxidative stress in *S. pombe* (Leong et al. 2014). Furthermore, recently, a lncRNA termed *HAX1* was identified as a *trans*-activator of cellulase expression in *T. reesei* (Till et al. 2018). Interestingly, all three of these lncRNAs (i.e. *SUT457*, *SPNCRNA.1164* and *HAX1*) have a positive regulatory impact on their target genes. However, also yeast lncRNAs with a repressing function in *trans* are known. For example, members of the class of the Xrn1 unstable transcript (XUTs), such as *TY1,* frequently mediate silencing of *trans*-located target genes (Berretta et al. 2008).

### The role of lncRNAs in cellular processes

Some lncRNAs can be categorized according to their functional roles in the yeast cells. *IRT1*, *RME2* and *RME3*, all of which acting on the regulation of meiosis in *S. cerevisiae*, are similar regarding their function and regulatory mechanisms. *IRT1* (*IME1* regulatory transcript 1) inhibits the expression of the downstream located and partially overlapping sense-oriented gene *IME1* (inducer of meiosis 1), thereby preventing erroneous germ cell differentiation and sporulation in haploids (van Werven et al. 2012). In cells grown in a haploid state, the meiosis-repressive transcription factor Rme1 (repressor of *IME1*) induces the production of the lncRNA *IRT1*. *IRT1* then mediates depositioning of repressive chromatin marks by recruiting the histone methyltransferase Set2 and the histone deacetylase Set3, which leads to a block of transcription initiation of *IME1*. Moreover, *IRT1* hinders binding of the transcription activator Pog1. Upon the onset of meiosis as a response to carbon source and nitrogen starvation in MATa/MATα heterozygous diploid cells, the expression of the Rme1 encoding gene is inhibited by binding of the diploid-specific a1-α1 repressor complex (Mitchell and Herskowitz 1986). This consequently shuts off formation of the nc transcript *IRT1* and allows expression of *IME1*. Anyway, for the switch from vegetative growth to entry into meiosis in diploids, another mechanism is dominating the regulation of *IME1* and *IRT1*. Under nutrient-rich conditions, PKA (protein kinase A) and TORC1 (target of rapamycin complex I) are produced in both haploid and diploid cells. Those factors are required for binding of the Tup1–Cyc8 complex to the *IME1* locus and hence direct inhibition of both *IME1* and *IRT1* when nutrients are available (Moretto and van Werven 2017). Synergistically, the mechanisms based on regulation by PKA/TORC and Rme1 allow tightly control of entry into meiosis only upon nutrient starvation and in heterozygous diploid *S. cerevisiae* cells exclusively.

Though less research has been done on the two other meiotic lncRNAs in *S. cerevisiae*, some parallels regarding their regulatory strategies can be noted. Like *IRT1*, *RME2* (regulator of meiosis 2) prevents germ cell differentiation in haploids and interferes with the transcription of its target gene *IME4* depending on the presence or absence of Rme1 (Hongay et al. 2006). Also, *RME3* (regulator of meiosis 3) represses the expression of its adjacent gene *ZIP2*, thereby preventing formation of the synaptonemal complex, which is required for chromosome pairing during meiosis (Gelfand et al. 2011). Both *RME2* and *RME3* are initiated downstream and transcribed in antisense orientation relative to their target genes, and both are interfering with transcription elongation rather than initiation (Gelfand et al. 2011). Furthermore, for both lncRNAs, a mechanism based on regulation of the chromatin status was postulated. However, there are not any details known.

A similar regulatory model as described for *IRT1* has also been postulated for the *SRG1*, a lncRNA that negatively interferes with the expression of the *SER3* gene under serine-rich conditions in *S. cerevisiae* (Martens et al. 2004).

**Fig. 1:**
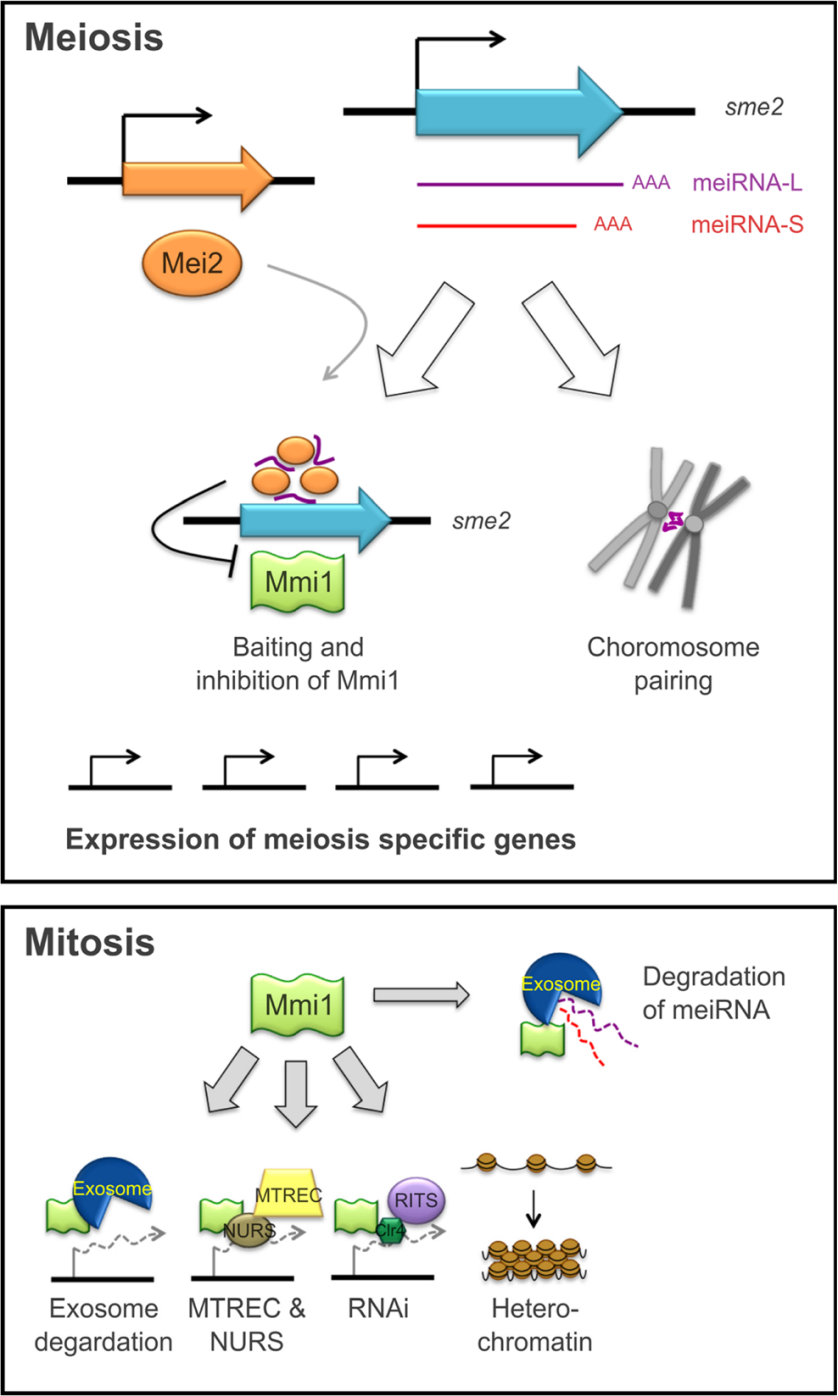
Regulation of meiotic gene expression in *S. pombe* by meiRNA. Two isoforms of meiRNA differing in length result from variation of the polyadenylation sites: meiRNA-L and meiRNA-S. The long version meiRNA-L has the more striking role in meiosis progression. Upon the onset of meiosis, meiRNA-L accumulates with its binding partner Mei2 at the *sme2* locus that governs baiting and inhibition of the key-silencing factor Mmi1. Thus, meiosis specific genes, which are destabilized by Mmi1 during mitosis, are stably expressed. Moreover, meiRNA-L mediates chromosome pairing during the meiotic prophase. During mitosis, Mei2 is not produced and meiRNA is destabilized by Mmi1-directed exosome degradation. Thus, Mmi1 is active and causes gene silencing by mediating exosome degradation, recruitment of the RNA degradation complexes MTREC and NURS, as well as the RNAi machinery (the histone methyltransferase Clr4 and the RNAi effector complex RITS) and by promoting heterochromatin formation

Interestingly, also in *S. pombe*, meiosis is controlled by a lncRNA termed meiRNA; however, its regulatory strategy is strikingly different. Compared to *IRT1* in *S. cerevisiae*, meiRNA is not involved in the induction of meiosis, but in meiosis progression (Watanabe and Yamamoto 1994) and also chromosome pairing (Ding et al. 2012). Two isoforms of the meiRNA (i.e. meiRNA-S and meiRNA-L) are transcribed from the locus and physically interact with RNA-binding protein Mei2 upon onset of meiosis (Watanabe and Yamamoto 1994). During meiosis progression, the meiRNA-Mei2-complexes accumulate at the locus, leading to the formation of the so-called Mei2 dot (Yamashita et al. 1998). This finally causes baiting and inhibition of Mmi1, a key-silencing factor associated with heterochromatin formation, RNAi and also Mmi1-mediated RNA degradation (Shichino et al. 2014). As a consequence, genes required for meiosis progression are stably expressed (Fig. 1). Interestingly, due to its ability to physically interact with Mmi1, meiRNA itself is a target of Mmi1-mediated RNA degradation in mitotic cells (Hiriart et al. 2012; Yamashita et al. 2012). However, during meiosis, meiRNA is supposed to be stabilized upon complex formation with Mei2 and sequestering of Mmi1 (Harigaya et al. 2006; Hiriart and Verdel 2013). Schematic illustrations of the mechanisms of the here presented lncRNA are provided in several review articles (Hiriart and Verdel 2013; Niederer et al. 2017; Yamashita et al. 2016).

Besides meiosis, a couple of lncRNAs involved in telomere synthesis and maintenance in yeast are known. This process is essential to protect chromosome ends from shortening and prevent erroneous repair initiated by the DNA-damage response (de Lange 2005). In *S. cerevisiae*, two sorts of telomere-associated lncRNAs have been described: *TERRA* (telomeric repeat-containing RNA) and the telomerase RNA *TCL1. TERRA* acts as a scaffold for telomeric DNAs and chromatin-modifying enzymes during telomere synthesis and regulates telomerase activity (Luke et al. 2008). It has been extensively reviewed elsewhere (Cusanelli and Chartrand 2015; Luke and Lingner 2009).

*TLC1* provides a platform for the formation for the telomerase complex itself and serves as a template for reverse transcription by Est2 (Zappulla and Cech 2004). A homologue to *TLC1* was also discovered in *S. pombe,* namely the telomerase RNA *TER1* (Leonardi et al. 2008). Some characteristics are shared between the two telomerase RNAs; however, *TER1* was found to be larger than *TLC1* and contains a higher number of invariant repeats compared to *TLC1* that is rather heterogeneous.

Gene silencing and broad heterochromatin formation is often associated with the degradation of unstable transcripts (reviewed in Tudek et al. 2015; Wu et al. 2012). This issue will be discussed in more detail in “lncRNAs with special properties”.

**Fig. 2:**
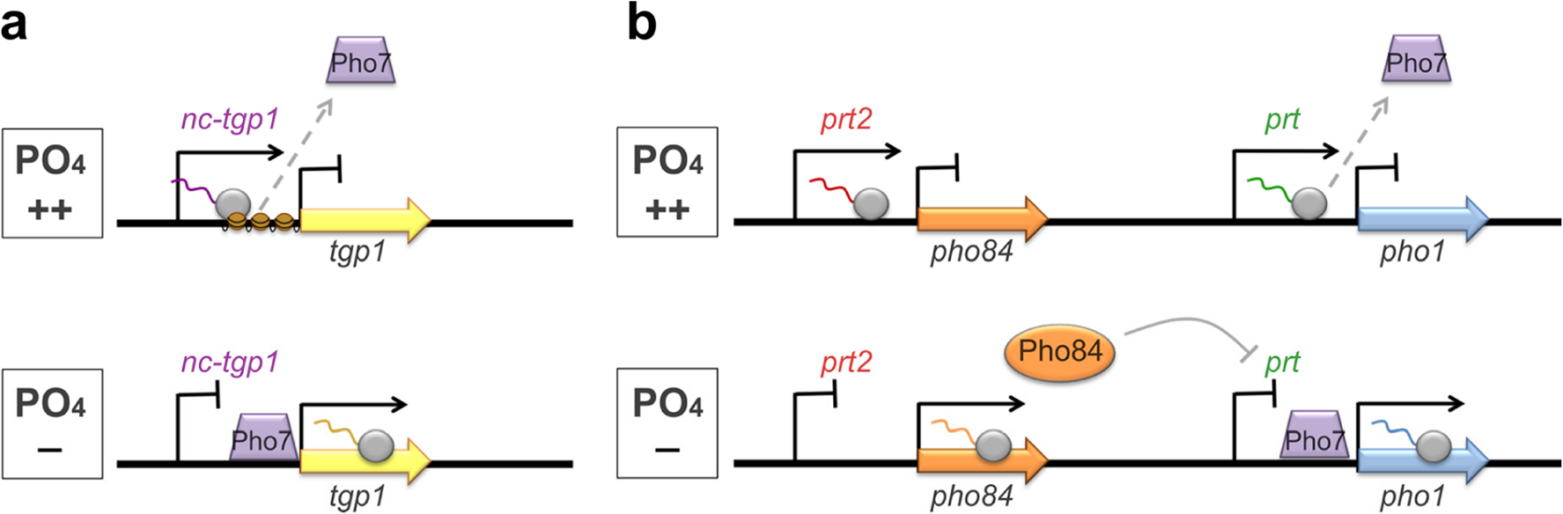
Response to extracellular inorganic phosphate in *S. pombe*. **a** Inversely correlated expression of *tgp1* and the lncRNA *nc-tgp1* in the presence or absence of phosphate. Under phosphate-rich conditions, the lncRNA *nc-tgp1* is transcribed and blocks the expression of its sense gene *tgp1* by modulation of the local nucleosome arrangement and promoting the dissociation of the central transactivator Pho7. Upon phosphate starvation, nc-tgp1 initiation is prevented, thus allowing binding of Pho7 and expression of *tgp1*. **b** Inversely correlated expression of *pho84* and the lncRNA *prt2* as well as *pho1* and the lncRNA *prt* in the presence or absence of phosphate. Under phosphate-rich conditions, the lncRNA *prt2* is transcribed and blocks the expression of its sense gene *pho84* by an unknown mechanism. Similarly, the adjacent lncRNA *prt* is transcribed and blocks the expression of its sense gene *pho1* by promoting the dissociation of the central transactivator Pho7. Upon phosphate starvation, *prt2* initiation is prevented; thus, Pho84 is produced and in turn acts as a repressor of *prt* transcription, finally resulting in the expression of *pho1*

Also, the response to phosphate starvation in *S. pombe* is regulated by a set of lncRNAs sharing some features. All of them are transcribed under phosphate-rich conditions and repressed upon starvation and all of them cause repression of their sense-oriented target genes in *cis* via transcription interference (Ard et al. 2014; Chatterjee et al. 2016; Garg et al. 2018). *Nc-tgp1* and *prt* have been investigated in more detail. In response to extracellular inorganic phosphate, the lncRNA *nc-tgp1* is produced and alters the nucleosome density at the promoter of its adjacent gene *tgp1* (transporter for glycerophosphodiester 1) (Ard et al. 2014). This results in dissociation of the central transactivator Pho7 and, thus, in a total shut-off of *tgp1* transcription (Fig. 2a).

A similar regulatory model was proposed for the regulation of *pho1* expression by the lncRNA *prt* (*pho1*-repressing transcript). Also in this case, *pho1* is repressed in response to *prt1* transcription under phosphate-rich conditions and expressed upon phosphate starvation, strictly depending on activation by Pho7 (Fig. 2b) (Chatterjee et al. 2016). Initially, another model for the regulation of *pho1* expression by the lncRNA *prt* was suggested. It is based on the recruitment of Mmi1 by the lncRNA *prt*, which results in depositioning of repressive chromatin marks and RNAi-mediated silencing (Shah et al. 2014). Yet, later research rather supports the concept of transcription interference by hampering binding of Pho7 (Chatterjee et al. 2016; Garg et al. 2018). Recently, the dissociation of Pho7 from the *pho1* locus as a consequence of *prt* transcription was reported to be governed not only by the lncRNA *prt* itself but also by RNA polymerase II (Pol II), depending on its phosphorylation status (Chatterjee et al. 2016). According to this model, Pol II moves towards the *pho1* promoter during progression of *prt* transcription and antagonizes binding of Pho7 close to the Poly(A) site of *prt*, thus resulting in the loss of *pho1* initiation (Fig. 2b). Changes in the phosphorylation status of Pol II are supposed to lead to prior termination of *prt* transcription, consequently resulting in de-repression of *pho1*. A likewise mechanism was also shown for *nc-tgp1* and its target gene *tgp1* (Sanchez et al. 2018). Both lncRNAs, *prt* and *nc-tgp1,* are controlled by Mmi1-directed exosome degradation and RNAi, as they harbour a cluster of DSR (determinant of selective removal) motives which are recognized and bound by the central silencing factor Mmi1. Yet, this mechanism is independent from the regulatory impact on their adjacent genes (Ard et al. 2014; Chatterjee et al. 2016).

Recently, a third lncRNA acting on the phosphate metabolism in *S. pombe* has been discovered, namely *prt2* (Garg et al. 2018). Like its two functionally related lncRNAs, *prt2* is transcribed upon phosphate starvation and governs repression of its adjacent gene *pho84* (Fig. 2b). Moreover, also for *prt2*, an impact of the phosphorylation status of Pol II on expression of its target gene was confirmed. Conclusively, a similar mechanism as described for *prt* and *nc-tgp* can be supposed. Interestingly, *prt2* was shown to effect the production of the proximal located lncRNA *prt* and its regulated gene *pho1*. Inactivation of *prt2* results in an upregulation of Pho84, which consequently leads to a downregulation of *prt* and finally a stimulation of *pho1* transcription (Garg et al. 2018).

## Regulatory strategies of lncRNAs

The simplest and probably most common regulatory strategy of lncRNAs is interference with the transcription of proximal located genes (reviewed in Kornienko et al. 2013; Vance and Ponting 2014). They can govern the expression of sense or antisense located genes in a repressing or activating manner by blocking the transcription machinery, modulation of the nucleosome arrangement and thereby provoking dissociation or binding of regulatory factors like transcription factors. Examples for a repressing effect of sense directed lncRNAs are *nc-tgp1*, *prt* and *prt2*, which have been described in the prior section.

Besides those, an interesting pair of lncRNAs is known that enables cell-cell adhesion during filament formation of *S. cerevisiae* cells in response to nutrient starvation, namely *ICR1* and *PWR1* (Bumgarner et al. 2009). They regulate the expression of their adjacent gene *FLO11* in a synergetic and sophisticated way. The current concept is a three-state model, comprising an activated, a repressed and a basal state (Bumgarner et al. 2012; Octavio et al. 2009). In the basal state, the lncRNA *ICR1* is produced and causes dissociation of the potentially bound activating factor Flo8 as well the repressing factor Sfl1 from the *FLO11* promoter. For activation of *FLO11* expression, Flo8 is bound and triggers the transcription of the antisense lncRNA *PWR1*, which acts as a suppressor of *ICR1* transcription. In the repressed state, Sfl1 binds to the promoter and mediates silencing by the recruitment of the histone deacetylase Hda1.

Another prominent example for transcription interference by lncRNAs is *SRG1*, which has been extensively reviewed (Hiriart and Verdel 2013; Niederer et al. 2017; Yamashita et al. 2016) and was briefly mentioned in the prior paragraph. It is transcribed under serine-rich conditions in response to activation by Cha4 (Martens et al. 2005) and mediates nucleosome depositioning at the *SER3* promoter depending on FACT, Spt6/Spn1 and Spt2 in order to repress the expression of *SER3* (Martens et al. 2004). As depicted in the prior section, similar mechanisms were described for *IRT1*, *RME2* and *RME3,* yet in this case, also an impact on the chromatin arrangement by directing depositioning of repressive chromatin marks was postulated.

The quite newly identified pHO-lncRNA forces nucleosome repositioning at the locus of the downstream located *HO* gene in *S. cerevisiae* (Yu et al. 2016). The regulated gene encodes the *HO* endonuclease, which is responsible for mating type interconversion during re-entering of the cell cycle after pheromone-dependent arrest in G1. Transcription of pHO-lncRNA is induced in response to the production of a pheromone (i.e. the α-factor) and causes nucleosome rearrangement and displacement of the factor SFB from the *HO* promoter. The loss of the activating signal from SFB results in a shut-off of *HO* expression and thus prevention of mating-type interconversion during re-entering into the cell cycle.

An exceptional mechanism for the regulation by a lncRNA was recently described for *SUT169* (Huber et al. 2016). Upon nutrient starvation, *SUT169* is transcribed and promotes the production of a sporulation-specific protein encoded by the *SPS100* gene. However, in contrast to other lncRNAs, *SUT169* does not activate the expression of it target gene, but it interferes with the transcription by supporting the production of a longer and more stable isoform of *SPS100*.

Another regulatory strategy followed by lncRNAs is depositioning of histone modifications and the recruitment of chromatin remodelling factors. One well-known example for this is the *GAL10* lncRNA. In *S. cerevisiae*, the expression of the *GAL* genes (i.e. *GAL1* and *GAL10*) is switched on in the presence of galactose and repressed in the presence of glucose to allow regulation of galactose metabolism. Under repressing conditions, *GAL10* ncRNA is produced and mediates di- and trimethylation of K4 and dimethylation of K36 on histone 3 by Set2 (Houseley et al. 2008). Those repressive chromatin marks are bound by Eaf3, which recruits the histone deacetylase Rpd3S, thus resulting in broad deacetylation and silencing of the whole *GAL* locus. In addition, H3K4me2 and H3K4me3 cause a delay in the recruitment of Pol II and TBP to the *GAL10* promoter (Geisler et al. 2012). Another lncRNA acting on the *GAL* genes is *GAL4* lncRNA. Here, more studies are required to understand its mechanism in detail (Geisler et al. 2012).

Further examples of lncRNAs effecting the chromatin organization are the mlonRNA from *S. pombe* and the *ncASP3* and the antisense lncRNA of the *CDC28* gene in *S. cerevisiae*, all of which are involved in stress response. The latter is induced by the stress-activated protein kinase Hog1 upon osmostress and supports translocation of Hog1 to the overlapping gene *CDC28* by bending the local genomic region into a loop (Nadal-Ribelles et al. 2014). This results in the recruitment of chromatin remodelers and thus activates the expression of the cyclin-dependent kinase 1 encoding gene *CDC28*.

Similarly, glucose starvation in *S. pombe* triggers the production of noncoding transcripts upstream of the *fbp1* gene, the so-called mlonRNAs (Hirota et al. 2008). These metabolic stress-induced lncRNAs trigger a switch of the chromatin configuration to an open state, which makes DNA more accessible for binding of Pol II and activators such as Atf1 (Hirota et al. 2008; Takemata et al. 2016). As a consequence, the *fbp1* gene (encoding the fructose-1,6-bisphosphatase) is expressed in the absence of glucose. The production of mlonRNAs in *S. pombe* is anticorrelated with antisense transcripts, which are produced from the *fbp1* locus under glucose-rich conditions (Miki et al. 2016). Compared to the *fbp1* mRNA, both mlonRNAs and their antisense transcripts were found to be prone to degradation by the nuclear exosome/Rrp6 complex (Galipon et al. 2013; Miki et al. 2016).

The lncRNA *ncASP3* in *S. cerevisiae* influences the expression of its target gene *ASP3* in a peculiar way. *ASP3* is regulated by nitrogen catabolite repression (NCR) and encodes asparaginase II, an enzyme performing hydrolysis of asparagine to aspartate and ammonium cations in response to nitrogen starvation (Dunlop et al. 1978). *ncASP3* is expressed in both cases, when nitrogen is available and upon nitrogen depletion, and maintains an open chromatin configuration at *ASP3* by mediating trimethylation of H3K4. Thus, *ASP3* is kept in an accessible status, allowing immediate expression upon the onset of NCR (Huang et al. 2010).

Another type of regulatory strategy of lncRNAs is the interaction with proteins. lncRNAs can attract proteins, affect their function or act as scaffolds for several factors. Well-described examples for this in yeast are meiRNAs, *TERRA* or telomerase RNAs. They have been presented in detail in the prior section. Also for the recently identified lncRNA *HAX1* in *T. reesei*, an interaction with the main transactivator of cellulase expression can be supposed as a sequence element rich in Xyr1-binding sites is present at its 5′ end (Till et al. 2018). To date, details regarding the regulatory strategy of *HAX1* have not been reported.

## lncRNAs with special properties

Some lncRNAs are unstable transcripts (reviewed in Garneau et al. 2007; Tudek et al. 2015). They can be destabilized by different cellular components; anyway, their degradation allows tight control of their regulatory impact. One type of unstable lncRNAs are CUTs, which are degraded in the nucleus by the exosome/Rrp6 complex (Davis and Ares Jr 2006; Wyers et al. 2005). Examples are the *PHO84* antisense transcript (Camblong et al. 2007) or *TY1* (Berretta et al. 2008). As described for *prt* and *nc-tgp1*, degradation by the nuclear exosome can also be triggered by Mmi1 (Harigaya et al. 2006). XUTs and SUTs in contrast are exported to the cytoplasm for processing. There, they might be destabilized by de-capping mediated by Dcp1 and Dcp2 (Garneau et al. 2007). XUTs then are degraded by the 5′-3′ exonuclease Xrn1 (Nagarajan et al. 2013) or the *S. pombe* ortholog Exo2 (Szankasi and Smith 1996), whereas SUTs are processed by the cytoplasmic exosome (Garneau et al. 2007). Several XUTs and SUTs were shown to be targeted by the nonsense-mediated decay pathway, which is a cytoplasmic surveillance mechanism for recognizing and discarding mRNAs with premature stop codons (Smith et al. 2014; Tudek et al. 2015; Wery et al. 2016). One example for XUTs is the *GAL10* lncRNA (Houseley et al. 2008). NUTs (Nrd1-undetermined transcripts) are not controlled by post-transcriptional degradation but are sensitive to prior termination of lncRNA transcription by Nrd1 (Schulz et al. 2013) or the *S. pombe* ortholog Seb1 (Mitsuzawa et al. 2003). All these types of unstable transcripts cause silencing of their target genes. The mechanisms of degradation described here arise as a control of pervasive transcription and often act as a protection from gene silencing throughout the life cycle (Wyers et al. 2005). Hence, one could question whether the production of these unstable transcripts is really an explicit mechanism or rather an unwanted side effect of exhaustion of transcriptome surveillance.

Most lncRNA have a repressing effect on their target genes. The few examples of fungal lncRNAs acting as activators comprise the *CDC28* antisense transcript, meiRNA, *SUT169, SUT457*, *SPNCRNA.1164* and *HAX1*. Yet, in fact, they hardly share any features: some act in *cis*, others in *trans*; they occur in different organisms and are involved in different cellular processes ranging from telomere control over sporulation to metabolism and stress response. Details regarding the regulatory mechanisms of *SUT457*, *SPNCRNA.1164* and *HAX1* are unknown. However, due to its outstanding role as the first characterized lncRNA in a filamentous fungus, we will further address to *HAX*1 in this chapter.

**Fig. 3:**
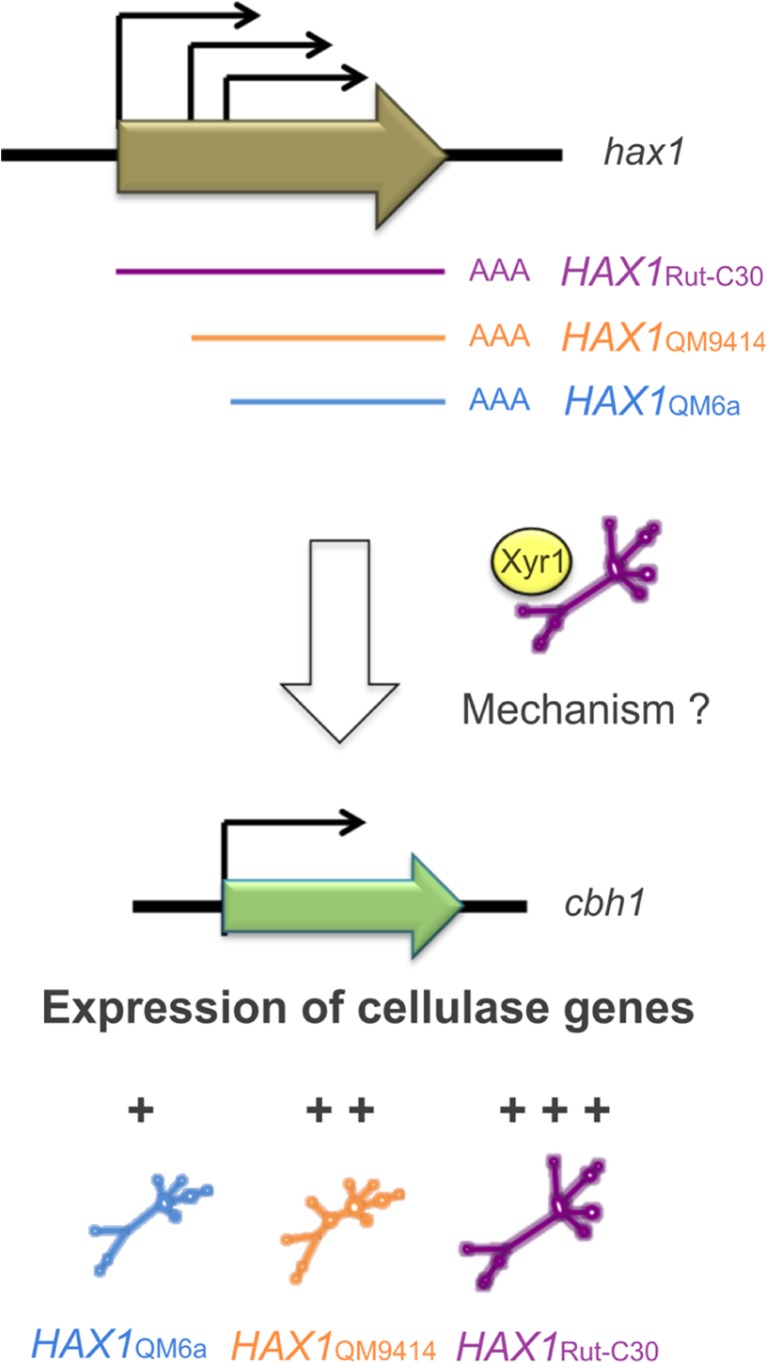
Regulation of cellulase gene expression by the lncRNA *HAX1* in *T. reesei*. Three isoforms of *HAX1* differing in length result from variation of the transcription start point in different *T. reesei* strains: *HAX1*_QM6a_, *HAX1*_QM9414_ and *HAX1*_Rut-C30_. They act as activators of cellulase expression. The longest version *HAX1*_Rut-C30_ has a higher impact on the cellulase activity compared to *HAX1*_QM9414_ and *HAX1*_QM6a_ (indicated by triple, double and single plus symbols, respectively). The regulatory mechanism of *HAX1* is unknown, yet an interplay with the main transactivator Xyr1 is supposed. For details, see text

*HAX1* was incidentally discovered by random integration of a marker cassette into the genome of *T. reesei* (Till et al. 2018). *T. reesei* is a saprophyte, growing on dead plant material (Klein and Eveleigh 1998). It secretes large quantities of cellulases and hemicellulases, which cause degradation of complex plant biopolymers (Ouyang et al. 2006). Especially, cellulases are commonly used for several processes meeting human regards; hence, *T. reesei* is widely applied as a high-yield producer of those enzymes in industry (Kubicek and Penttilä 1998). The lncRNA *HAX1* was identified as an activator of cellulase expression (Till et al. 2018). Thus, *HAX1* has a pivotal role, both as a player in the complex regulatory network of cellulase expression and as a potential biotechnological tool for the improvement of enzyme production. While details on the regulatory strategy are not reported yet, the presence of a sequence element rich in Xyr1-binding sites suggests an interplay with the main transactivator of cellulase and xylanase expression, the Xylanase regulator 1 (Xyr1) (Rauscher et al. 2006; Stricker et al. 2006). One special feature regarding *hax1* expression is the strain-specific variation of RNA length (Till et al. 2018). Interestingly, much shorter versions of *HAX1* are present in the *T. reesei* wild-type strain than in moderate or in high cellulase overproduction strains. Overexpression of the three identified *HAX1* versions in the wild-type strain led to an increase in cellulase activity, depending on RNA length. The shortest version only slightly improved cellulase expression, whereas the longest version led to the strongest increase in cellulase activity (Fig. 3). Conclusively, a direct link between *HAX1* length and its regulatory role can be supposed.

Another example for the variation of transcript length in fungi is the above-discussed meiRNA in *S. pombe*. Two isoforms of this lncRNA are produced: the 0.5 kb meiRNA-S and the approximately 1.2 kb meiRNA-L (Watanabe and Yamamoto 1994). The production of these isoforms depends on the presence of different polyadenylation sites, initiating transcription termination (Fig. 1). In contrast, the differences in *HAX1*-length result from variation of the transcription start point (Fig. 3). Also, the transcription of meiRNA-L is initiated at a more or less defined region rather than a certain position. However, in literature, the variety of transcripts of meiRNA-L slightly differing in length is specified as one RNA species. A vague definition of transcription start- and end points is not uncommon for lncRNAs. Heterogeneity in 3′ and 5′ ends was also stated for other lncRNAs such as telomerase RNAs (Dandjinou et al. 2004; Leonardi et al. 2008). However, the special feature of meiRNA- and *HAX1* isoforms is that they have different regulatory impacts. Similar to *HAX1* and against initial assumptions, the long isoform meiRNA-L turned out to have a more striking role in meiosis progression than meiRNA-S, both for the attraction of Mmi1 (Shichino et al. 2014) and for chromosome pairing (Ding et al. 2012). Anyway, strain-specific variation of RNA length remains an exceptional strategy solely described for *HAX1* so far.

As the functionality of lncRNAs commonly depends on their distinct folding, grouping of lncRNAs based on their structural features might also be a promising way to go. This would also allow the separation of certain species such as unstable transcripts (e.g. CUTs, XUTs, NUTs) or ribonucleo-proteins (e.g. telomerase RNAs). In human, attempts for the prediction of lncRNAs based on structural mappings were presented (Washietl et al. 2005a; Washietl et al. 2005b). Also in yeast, studies on genome-wide profiling of the secondary structures of ncRNAs were performed (Kertesz et al. 2010). Yet, more research would be needed to better assess the potential of this feature for the classification of lncRNAs.

## Summary and conclusion

lncRNAs emerged as a heterogeneous group of noncoding transcripts involved in a variety of regulatory processes in eukaryotes. Initially, they were discovered in human; meanwhile, several lncRNAs have been identified in fungi. Some examples of yeast lncRNAs are well known and thoroughly described in literature, whereas others have just recently been identified and need to be investigated in more detail. lncRNAs are very diverse regarding their features, functions and regulatory strategies. They are involved in cellular processes such as metabolism and stress response, cell cycle control (e.g. meiosis), telomere maintenance or gene silencing. Their regulatory mechanisms range from transcription interference and local nucleosome rearrangement, over depositioning of histone modifications and chromatin remodelling, to physical interactions with several factors, acting as a scaffold or bait. Some lncRNAs might be distinguished upon certain characteristics (e.g. CUTs, XUTs, NUTs), and others acting on the same cellular process share a similar mode of action. However, in general, classification of lncRNAs is a challenging task. One possible property for categorizing lncRNAs in the future might be their higher ordered structure. Unfortunately, at the current state of knowledge, not enough data are available on this issue.

In addition to yeast lncRNAs, recently, the first functional characterization of a lncRNA was reported for a filamentous fungus, namely *T. reesei*. It was presented as a potential tool for strain improvement and the industrial exploitation of *T. reesei*. Similarly, a specific use of yeast lncRNAs for industrial purposes might be considered. Both *S. cerevisiae* and *S. pombe* are commonly applied for biotechnological production processes. Hence, targeted intervention in processes influenced by lncRNAs represents a promising strategy for process optimisation. Ongoing research and steadily improving technologies provide the basis for uncovering yet unknown mechanisms of lncRNAs, their biotechnological applications and the identification of new candidates in yeasts and higher fungi.
